# Partner intimate touch is associated with increased interpersonal closeness, especially in non-romantic partners

**DOI:** 10.1371/journal.pone.0246065

**Published:** 2021-03-10

**Authors:** Nicole Prause, Greg J. Siegle, James Coan

**Affiliations:** 1 Liberos, Los Angeles, California, United States of America; 2 Department of Psychiatry, University of Pittsburgh School of Medicine, Pittsburgh, Pennsylvania, United States of America; 3 Department of Psychology, University of Virginia, Charlottesville, North Carolina, United States of America; Universita degli studi di Padova (Padua University), ITALY

## Abstract

Relationship closeness promotes desirable health outcomes. Most interventions to increase relationship closeness are verbal, which may not suit all couples. We consider whether Orgasmic Meditation (OM), a structured, partnered, largely non-verbal practice that includes genital touch, also increases relationship closeness. We hypothesized that OM would increase feelings of closeness for both romantic and non-romantic partners. This is important, because intimate touch with non-romantic partners is commonly considered deleterious by clinicians, which may inadvertently increase feelings of shame. Dyads (n = 125) reported their feelings of closeness before and after OM. Approximately half of the participants were romantic partners, while the other half only engaged in OM together (non-romantic). Closeness after OM increased on average across participants. Non-romantic dyads increased self-other overlap more than romantic dyads. These data support that a partnered, largely non-verbal practice is associated with increased feelings of closeness in the moment, including for individuals who are not in a romantic relationship.

## Introduction

It may be intuitive to think that sexual touch could increase relationship closeness. The literature on romantic relationships is fairly uniform in this regard, whereas the literature on the average effects of consensual sexual touch in non-romantic relationships is more mixed. Here, we consider whether this variation may be mitigated by context by examining uniformity of effects on relationship closeness across romantic versus non-romantic relationships in a specific safe practice involving structured, sexual touch devoid of many of the other uncontrolled aspects of usual sexual interactions, such as professing love.

In romantic relationships, more frequent sexual intercourse has been associated with a variety of nominally positive variables that could be proxies for relationship closeness such as higher marital satisfaction, sexual satisfaction, body image [1], emotional satisfaction [2], life satisfaction [3], and relationship stability [4, 5]. Of course, intercourse frequency is only one way to characterize sexual activity. These associations were more robust when the sexual repertoire was more broad [6], orgasms were more consistent [7], the sex was satisfying, and the relationship was warm [8]. Most such effects reported are from surveys of individuals within a couple, not including both members of the couple. The match of desired sexual frequency strongly impacts the sexual satisfaction of the individual [9]. Dynamic dyad relationships rapidly become more complicated. For example, a couple with discrepant desire still reports higher sexual satisfaction when they perceive the sexual activity they do have to feel more emotionally connected [10]. In general, more sex in romantic relationships is associated with a wide variety of positive health indicators. That said, other data already suggest limitations on the benefits of romantic partner sex. Sex more often than weekly does not appear to improve benefits [11] and sexual frequency increased in response to instructions in a study actually was detrimental to happiness and sexual satisfaction [12].

Research outcomes likely related to relationship closeness appear even more mixed for sexual activities with a non-romantic partner [13]. Sex with non-romantic partners often includes some intimate, affectionate behaviors thought to characterize exclusive, romantic relationships, such as extended foreplay, eye gazing, cuddling [14], and seeking emotional gratification [15]. Yet the outcomes vary across studies. Young women tend to experience more distress and drug use following casual sexual encounters, while young men appear unaffected [16]. In sexual relationships that are non-exclusive and non-committed, sexual satisfaction and communication tends to be poorer than in exclusive committed relationships; however, in casual relationships couples also spend more time having sex and communicating more about outside partners [17]. Sometimes the positive or negative effects of sexual activity with non-romantic partners depend on the status of the individual. For example, casual sexual encounters between people with no relationship expectations, improved depression symptoms four months later in depressed individuals, but were associated with increased depressive symptoms in those who were not depressed at the first assessment [18]. Those who participate in hook-up culture are often described as at-risk of sexual assault and perpetration [19] and severe mental health problems [20]. Descriptions of these encounters from individuals display these mixed responses. From Tholander and Tour [21] a young woman described “He was like really gave compliments and he well, we were kind of spooning and he was very cuddly afterwards, even though we just kind of had sex with each other. And both of us knew that I would leave the next morning, probably.”

From these data, it could appear that sex with a romantic partner may largely lead to features associated with increased relationship closeness whereas evidence for such effects of sex with a non-romantic partners is more mixed. Yet, the examined interactions may differ on many variables other than the type of relationship between the partners, and women carry a higher risk burden interacting with a non-romantic partner. Women with more casual sex partners experience more sexual coersion and sexual assault than women who have fewer casual sex partners [22]. Women who engage in casual sex are judged to be less intelligent, less competent, “promiscuous”, and have poorer mental health relative to men who accept casual sex [23]. Women are expected not to desire non-romantic sexuality [24]. A review of this literature concluded that the context of the casual sex, rather than the sex itself, was likely the more common cause of this variability [25]. For example, sex with non-romantic partners is much more likely to occur following alcohol consumption [26], which may be potentiating the other negative outcomes such as neglecting to use a condom. The current study thus investigates whether, if we control contextual features, a sexual interaction would be as beneficial for non-romantic partners as romantic partners.

### Feeling connected

Many of the benefits thought to accrue following sexual interactions could be described as arising from feeling connected to another person. The extent to which concepts of “self” and “other” overlap is sometimes described as relationship “closeness” [27], and we use these interchangeably. Self-expansion in romantic relationships may also occur by mechanisms other than inclusion of other in the self, such as the novel experiences offered by the interests of their partner. Self-expansion in relationships is proposed to come, in part, from approach motivation that arises pursuing pleasure [28]. Having experiences with a partner that are intense, especially when they are experienced positively, are strongly associated with self-expansion [29]. However, self-expansion is not due merely to pleasantness, positive affect, arousal, or avoidance motivation [30]. Identifying methods to increase self-expansion is desirable, because self-expansion is associated with a number of health benefits.

Higher inclusion-of-other-in-self in a romantic relationship promotes a variety of health and well-being outcomes [31], including reducing the effort required of either individual in the dyad as burdens become more shared [32, 33]. Also, closer relationships result in greater intentions to help the partner [34], higher average relationship well-being [35], greater compassion [36], positive trait attributions to the other [37], and lower feelings of loneliness [38]. Increasing relationship closeness in-the-moment improved couple’s problem resolutions [39], increased helping [40], improved physiological reactivity to stressors [41], lower perceived stress [42], increased empathy for another person [43], improved self-control [44], and promoted positive emotions [45]. Self-other overlap also has been characterized in other forms of close relationships, including friendship [46] and kinship [47]. Self-other overlap is further extended in the current study to characterize intimate-but-nonromantic relationships.

### Sexual intimacy in non-romantic dyads

Relationship closeness is likely to be moderated by dyad-wise individual differences. The nature of the dyad has proven important to predicting the outcome of other types of interactions. Higher intensity experiences appeared especially effective generating attraction with a stranger in the laboratory [48]. Heterosexual male participants having their leg caressed by a novel female responded with greater somatosensory cortex activity than when they believed they were having their leg caressed by a novel male [49]. In fact, these somatosensory differences by caresser gender emerged as soon as the touch was anticipated (before it occurred), strongly suggesting that context may modulate the effects of touch more strongly than the nature of the touch itself. Although holding someone’s hand is an effective method for reducing threat perception, this effect is largest for happily married couples and those who perceive themselves to have a generally high degree of access to social resources [50, 51]. We suggest that relationship status (romantic or non-romantic) may be a salient moderator in the degree of self-other overlap is familiarity [33]. Getting to know another person early in a relationship is thought to propel particularly rapid self-other overlap, which is experienced as pleasant [52].

Specific hypotheses differentiate these possibilities. Based on the reviewed literature, sexual interaction with non-romantic partners may increase or decrease self-other overlap in non-romantic partners, while it should increase self-other overlap in romantic partners. We test these alternative hypotheses in a highly structured practice called Orgasmic Meditation (OM). OM was ideal for testing the effects of relationship status, because it reduces variability uniquely for women due to fear of assault, disease, or pregnancy. OM only includes providing sexual stimulation to women (described below), and thus this study cannot address gender effects.

### Sexual intimacy in the laboratory

OM was used to isolate the effects of genital touch. OM is similar to sensate focus exercises [53], a common form of sex therapy, in its approach. Both include non-demand, goal-free pleasuring that is structured by time limits and areas of physical stimulation. Where sensate focus is typically used to address pathology, OM is typically used as a wellness practice. OM is a standardized protocol that is, in many ways, ideal for laboratory study. It has an inherent structured consent process that features unidirectional, genital touch. OM partners do not need to be romantic partners. Further, OM provides biohazard management and minimal movement between partners, which makes it very useful for laboratory assessments. The practice’s primary goal of “just experiencing” physical sensations minimized demand characteristics. Fifteen minutes of the practice is indirect, manual stimulation of the clitoral shaft, which is the preferred area for female genital stimulation [54]. As a first experiment using OM, we were concerned that recruiting couples completely new to OM and attempting to teach them OM would introduce too many confounds. These confounds include a lack of familiarity with the procedure, anxiety about the possibility of experiences during OM that might recall sexual trauma, and a lack of practice expressing continual consent or feedback to the partner during OM. Thus, while OM-naive couples could provide different information, we did not feel it was appropriate to recruit OM-naive couples for this first experimental study. Details of the practice and recruitment requirements appear in the Methods section below.

Regarding how participants understand the experience, the nature of OM may involve appraisal/meaning-making processes about the experience that impact views of self-other overlap. For example, the process of appraising a situation or cue as sexual varies by individual [55]. Even when a cue seems explicitly sexual, such as vaginal engorgement, this arousal is not consistently experienced as sexual [56]. If a person is seeking and allowing their clitoris to be stroked, they might infer some minimal level of physical and emotional safety exist. These safety signals alone might signal a closer relationship within the dyad that increase feelings of closeness. Such effects are suggested in related research, which demonstrated that a sexual relationship with an attractive partner was appraised as closer during fertile times of the menstrual cycle [57]. Relatedly, relationship closeness might be inferred to reconcile dissonant interpretations, such as that the partner might not be trustworthy. This appears similar to therapies that help individuals structure their sexual identity to resolve cognitive dissonance [58]. Couples who are concordant for viewing sex films (both view or neither view) also report greater self-other overlap, likely reflecting shared values and communication not challenged by negative interpretations of solo sex film use [59]. Finally, interpreting the relationship as close could be seen as a product of misattributed arousal processes [60], in which participants mistake arousal due to genital touch for arousal due to interpersonal closeness. Misattributed arousal reliably affects choice behavior [61].

A second mechanism that could associate OM with relationship closeness are broader aspects of the interaction not dependent on touch. The OM practice is modeled on features of assertive communication, demonstrations of caring, and dependable interaction. Such dependability and care are considered important aspects of supportive relationships, such as between a therapist and client [62]. Partnered yoga is similarly described as nurturing and depending on stable relationship qualities that allow working together [63]. Factors embodied in the OM practice, such as compassionate goals [64] and lack of controllingness [empowerment by the strokee, 65], also predicted increased relationship quality longitudinally.

Participants reported relationship closeness and sexual arousal before and after a single OM with a partner who was, or was not, a current romantic partner. Thus, as noted above, to the extent that the dysfunction predictions describe effects of genital stimulation, OM might not change or even decrease relationship closeness in non-romantic partners. The functional (e.g., self-expansion) hypothesis of pleasant partnered events predicted that non-romantic dyads should experience similar increases to romantic dyads. We also realize that sexual arousal is likely to be higher with a non-romantic partner, because the interaction would be more novel. We explicitly test whether sexual arousal varied as a function of partner type. Our hypothesized interaction is that sexual interaction will increase self-other overlap in all dyads, and interact with partner status (romantic or non-romantic).

## Methods

### Participants

Participants comprised 125 couples (N = 250) who had practiced OM at least 10 times in their lifetime, so that they had a general familiarity with the practice. They were recruited through both social media advertisements (Twitter, Facebook) and by word of mouth, including sharing on OM listservs. Participants had to self-report as being free from a history of neurological diagnosis (e.g., stroke, MS) to allow interpretation of neural measures (not analyzed in this manuscript), and self-reported to have normal or corrected-to-normal vision to be able to read from computer screens.

Participants also had to have an OM partner who also would qualify and participate, given the intimate nature of the intervention. They were required to have completed at least one OM together prior to the study to avoid multiple potential issues associated with novel partner sexual interactions as a function of the study (e.g., a known level of compatibility). However, this also meant that the “non-romantic” partners would not be entirely novel to one another; this work does not address first-time encounters. Each member of the dyad was screened independently by phone and provided separate written, informed consent to participate. Each participant agreed to abstain from any alcohol or recreational drug use in the 24 hours prior to participating. The laboratory was mobile (see below), so participants were tested in private settings that included apartments, office, and event spaces in Los Angeles, San Francisco, and New York.

### Inclusion of Other in the Self (IOS) scale

The IOSS is a rating thought to reflect interpersonal closeness [27]. Two circles are labelled as “self” and “other”. The circles begin as non-overlapping and progressively overlap until the two circles overlap almost entirely. Participants are instructed to select which of the seven images best portrays their relationship with their OM partner before beginning, then after, the OM. Support for the IOSS convergent and divergent, such as from community connectedness [66], validity has been demonstrated [27]. The IOSS discriminates between relationship types, such as friendships having less self-other overlap than engaged-to-marry dyads [67]. Important for state measures, the test-retest reliability of the IOSS was low [68].

IOSS has been related to a number of other indicators of interpersonal closeness. For example, the higher the IOSS scores, the more reactive the anterior cingulate cortex was to the errors of the close other [69]. In fact, the IOSS has been associated with many constructs relevant for pair bonding [70]. Experiences of self-expansion with a romantic partner predicted later, higher sexual desire for that partner [71]. The single-item IOSS also is associated negatively with relationship uncertainty, and relational turbulence [72].

### Sexual arousal experienced

Participants rated the level of “sexual arousal” (0-“Not at all”, 7-“Extremely”) that they felt. Rated sexual arousal was the primary dependent variable. This rating approach was first used by Heiman and Rowland [73] and is a common approach used to study felt sexual response in men and women. Rated sexual arousal tends to converge strongly with genital response in men, although the convergence is more variable in women [74]. Sexual arousal ratings are uniquely elevated to sexual stimuli [75]. Also, felt sexual arousal is prioritized over physiological measures in making clinical judgments of sexual problems, especially for women [76]. Finally, physiological sexual arousal that does not reach conscious awareness through attention is not expected to influence sexual function or behaviors.

### Experiences in close relationships: Attachment

Adult attachment has been widely studied as it relates to perceptions of closeness [77]. The Experiences in Close Relationships scale was designed to measure attachment feelings for romantic relationships in general [78]. This 36-item scale requests participants to rate items from 1 (“Strongly disagree”) to 7 (“Strongly agree”) with respect to “how you generally experience relationships, not just in what is happening in a current relationship”. Example items include “I feel comfortable depending on romantic partners” and “I do not often worry about being abandoned”.

The scale is thought to reflect two underlying dimensions of attachment: avoidance and anxiety [79]. Test-retest reliability of the scales after one month was high [80]. Scale convergent validity, such as with basic psychological needs satisfaction [81] predictive utility, such as for daily interactions with romantic partners [82]. The scale is used in this study to characterize the closeness of participant relationships. Cronbach’s alpha in the original study were .88 and .92 for anxiety and avoidance scores, respectively [78]. In our sample, Cronbach’s alpha was .92 (95% CI +/- .91 to .94) and .92 (95% CI +/- .90 to .93), respectively.

### Partner type

OM does not require that those practicing be romantic partners. Each individual privately answered the question on a computer “How would you describe your relationship with the person you are doing OM with today?” Response options included (1) Male/Female who also is my primary romantic partner, (2) Male/female who is a previous OM partner, but not my primary romantic partner, (3) Someone I am considering as a potential romantic partner, or (4) Other.

### Orgasmic Meditation

Orgasmic Meditation (OM) is a practice where partners engage in a practiced series of consent, safety signals, and 15 minutes of manual genital stimulation. The only goal is to feel sensations. The partners are referred to as “stroker”, the person (any gender) providing the manual stimulation, and “strokee”, the woman receiving the stimulation. The practice begins by laying a series of blankets and pillows on the ground to the comfort of the couple. These are structured to support the strokee lying prone with the stroker sitting comfortably to her right side with their legs intertwined. The strokee removes her shoes and removes or lifts bottom clothing to be bare from the waist down. The stroker removes his or her shoes. Socks may be worn for comfort. The strokee places her feet together to let her knees fall apart, comfortably supported by pillows. The stroker is able to clearly see the strokee’s vulva and reach her vulva with both hands without physical strain.

Next, the stroker announces that he or she is about to rest their hands on the strokee’s thigh. The stroker then briefly describes the appearance of the woman’s vulva using value-neutral terms, such as its shape, color, or texture. The strokee acknowledges this observation, typically by responding “thank you”. Then, the stroker dons gloves of agreed material (e.g., nitrile) to provide a physical safety barrier, regardless of the status of the couple (e.g., romantic dating partners would still use gloves). The stroker applies lubrication to the left index finger and right thumb. At this point, the stroker announces that he or she is about to touch the strokee’s genitals. The stroker places the right thumb at the introitus, not inside the vagina, where it remains throughout the stimulation period. The purpose of this placement is to aid the stroker to feel contractions or other movement. The stroker places the left hand with the thumb holding back any clitoral hood and the left index finger stroking beside the clitoral shaft.

The stroker then strokes the upper left area of the strokee’s clitoris for 13 minutes. The speed, pressure and other characteristics of the strokes are adjusted throughout. The strokee may request adjustments at any time, which are always followed by the stroker, and are acknowledged by the stroker saying “thank you”. The stroker might offer adjustments prior to making them at any time, which the strokee may accept or decline. At 13 minutes, the stroker typically announces “two minutes” (here, timed and announced by computer). At the end of two more minutes, the stroker covers the vulva with both hands with gentle pressure, allowing the vulva to close. The stroker uses a clean washcloth to wipe up once over the vulva to remove any fluids. The strokee sits up. The stroker and strokee take turns describing a concrete, bodily sensation (e.g., temperature, vibration, location) that occurred during stroking. The gloves and washcloth are discarded.

Throughout this practice, safety and consent is maintained by a number of practices. The structured practice means initial consent is understood to apply to each step that follows, although this can be revoked by either partner whenever desired. Additional contact, such as hugging, are prohibited during OM. Strokees learn, and are encouraged, to provide verbal instructions to the stroker if they experience any discomfort or prefer a change. Further, communication is explicitly informational and never complimentary. Sexual expressions or requests are also not allowed. Vocalizations (e.g., moaning, panting, etc.) outside of direct verbal communication are supported and common during OM, but not required. Physical climax may happen, but is not a goal of the OM practice.

### Procedure

All study procedures were approved by the University of [blinded for review] Institutional Review Board. Volunteers were contacted by phone and screened for inclusion criteria (see Participants). Each identified their intended partner by name, who volunteered independently and also provided that person’s name. They were scheduled for one, three-hour session in a private environment. After providing informed consent, they completed a series of questionnaires assessing demographics, sexual history, experience with OM, mental health, emotional attachment, and current feelings of closeness to their OM partner, sexual arousal, and emotions. They then donned equipment for assessment of electroencephalography (a measure of electrical brainwave activity). They completed a series of three computer tasks, one assessing their emotional responsivity, a persistent vigilance task, and a paced serial addition task. Results of the physiology and tasks will be reported elsewhere.

Afterwards, they completed one OM in a private room while their biological signals were monitored using additional physiological monitoring equipment. The couple set up their space as desired for the OM. The experimenter attached biological recording devices designed to not restrict movement. The strokee removed their clothing. The stroker advanced the OM by pressing the “space” on a keyboard beside him or her on the floor. The experimenter was outside the closed space during the OM.

After the OM, participants completed the same computer tasks again. They then answered questions about their response during the OM and their current feelings. Participants were offered the opportunity to ask any questions that they had. Each participant received $25 cash. Data were anonymized by eliminating all links with their Informed Consent. No one withdrew during the session.

Finally, an exploratory analysis was conducted. It may be that the close interaction of OM improves relationship quality in the moment (as may happen in a chance sexual encounter) and may not translate to longer term relationship quality. Data were collected regarding how often participants practiced OM in the typical month, which allowed an exploratory test of how long the effects observed might last. Participants could indicate practicing OM from 1 (Not once) to 7 (Most day or every day).

### Data analysis

A linear mixed model was used [lme4, 83] to test whether changes in closeness (or sexual arousal) around OM interact with the nature of the relationship of the dyad. Specifically, a mixed model of the closeness rating predicted by time reported (Pre OM, Post OM) was compared to the same/nested model adding the interaction term of partner type and time reported. Model fit was compared by chi-square test from ANOVA. Effect size was estimated as pseudo-R^2^ values [84].

Recruitment initially called for 117 dyads to power physiological measure analysis. High data loss (40 dyads) was estimated due to a highly novel protocol, so 157 dyads initially were targeted. This would reach a power 1-**β** = .8 moderate magnitude condition-related associations of Cohen’s d = .23. Ultimately, recruitment spread across three major cities to reach N = 125 couples at the end of the scheduled recruitment period. Recruitment was terminated at that time, because data retention was better than our initial estimates. Dyads who indicated that they were considering dating their OM partner (n = 13) or indicated the relationship was “other” (n = 25) were excluded from all analyses.

## Results

As shown in Table 1, participants were primarily mixed-gender dyads in their forties. OM partners who participated with their romantic partner were more likely to have a real-life romantic partner ***χ***^2^(df = 1) = 125.6,*p* < .001,*w*^*2*^ = .80. That is, some non-romantic dyads had romantic partners with whom they did not participate in this study. Given some low cell counts and the ability to select more than one identity, statistical tests were not performed on ethnic categories. Those who came to OM with a non-romantic partner reported more anxious *t*(*df* = 192) = 2.8,*p* = .006,*d* = .39 and avoidant *t*(*df* = 192) = 2.8,*p* = .006,*d* = .49 attachment than those who came to OM with a romantic partner. While not hypothesized, interactions of closeness with these attachment differences may be of interest. Using a linear model, the attachment scale scores were used to predict self-other overlap after OM while controlling for self-other overlap before OM. The addition of the attachment score term did not significantly increase the fit of the model for avoidance or anxiety attachment.

**Table 1:** Participant information by type of Orgasmic Meditation partner.

Variable	N*(n = 210)		Romantic (n = 106)		Non-romantic (n = 104)	
	Count	Percent	Count	Percent	Count	Percent
Female	111	52.9%	53	50.0%	58	55.8%
Employment						
Full time for pay	133	63.9%	64	61.5%	69	66.3%
Part time for pay	45	21.6%	26	25.0%	19	18.3%
Looking for paid work	15	7.2%	6	5.8%	9	8.7%
Not working or looking	15	7.2%	8	7.7%	7	6.7%
Have romantic relationship*	126	60.0%	103	97.2%	23	22.1%
Sexual orientation (self-id)						
Heterosexual	155	73.8%	75	70.8%	80	76.9%
Homosexual	0	0%	0	0%	0	0%
Bisexual	34	16.2%	20	18.9%	14	13.5%
Asexual	0	0%	0	0%	0	0%
Queer	8	3.8%	3	2.8%	5	4.8%
Something else	11	5.2%	7	6.6%	4	3.8%
Childhood sexual assault	30	14.3%	12	11.3%	16	15.4%
Climax during OM today						
Yes	24	11.4%	12	11.3%	12	11.5%
Unsure	10	4.8%	6	5.7%	4	3.9%
No	174	82.9%	87	82.1%	87	83.4%
Ethnicity**						
Indian	6	2.9%	4	3.8%	2	1.9%
Asian	24	11.4%	12	11.3%	12	11.5%
Pacific Islander	6	2.9%	3	2.8%	3	2.9%
Black	18	8.6%	9	8.5%	9	8.7%
White	164	78.1%	81	76.4%	83	79.8%
Education						
High school or less	7	2.8%	4	3.3%	3	2.5%
Some college	43	17.6%	25	20.5%	18	14.7%
College grad	120	49.1%	58	47.5%	62	50.8%
Masters grad	51	20.9%	26	21.3%	25	20.5%
More than masters grad	23	9.4%	9	7.4%	14	11.5%
Non-users of sex films	124	46.4%	33	12.3%	91	34.0%
	**Average**	**SD**	**Average**	**SD**	**Average**	**SD**
Age	41.9	11.6	42.0	12.2	41.9	11.0
Attachment style***						
Anxiety	3.3	1.2	3.1	1.0	3.5	1.3
Avoidance	2.8	1.0	2.5	0.9	3.0	1.1
Depressive symptoms	6.3	4.4	5.5	3.5	6.7	4.6

*Those who indicated their OM partner was a potential romantic partner or “other” were excluded from all analyses and this table.

** Does not sum to 100% due to ability to identify with more than one.

*** Higher scores are more avoidant; Avg from Andersen & Leibowitz (1978) Same sex: men = 26.4, women = 21.7; opposite sex: men = 12.9, women = 14.9.^Quick Inventory of Depressive Symptomatology includes 16 items for a sum total score that ranges from 0 to 27 with higher scores indicative of more difficulty.

**Fig 1:**
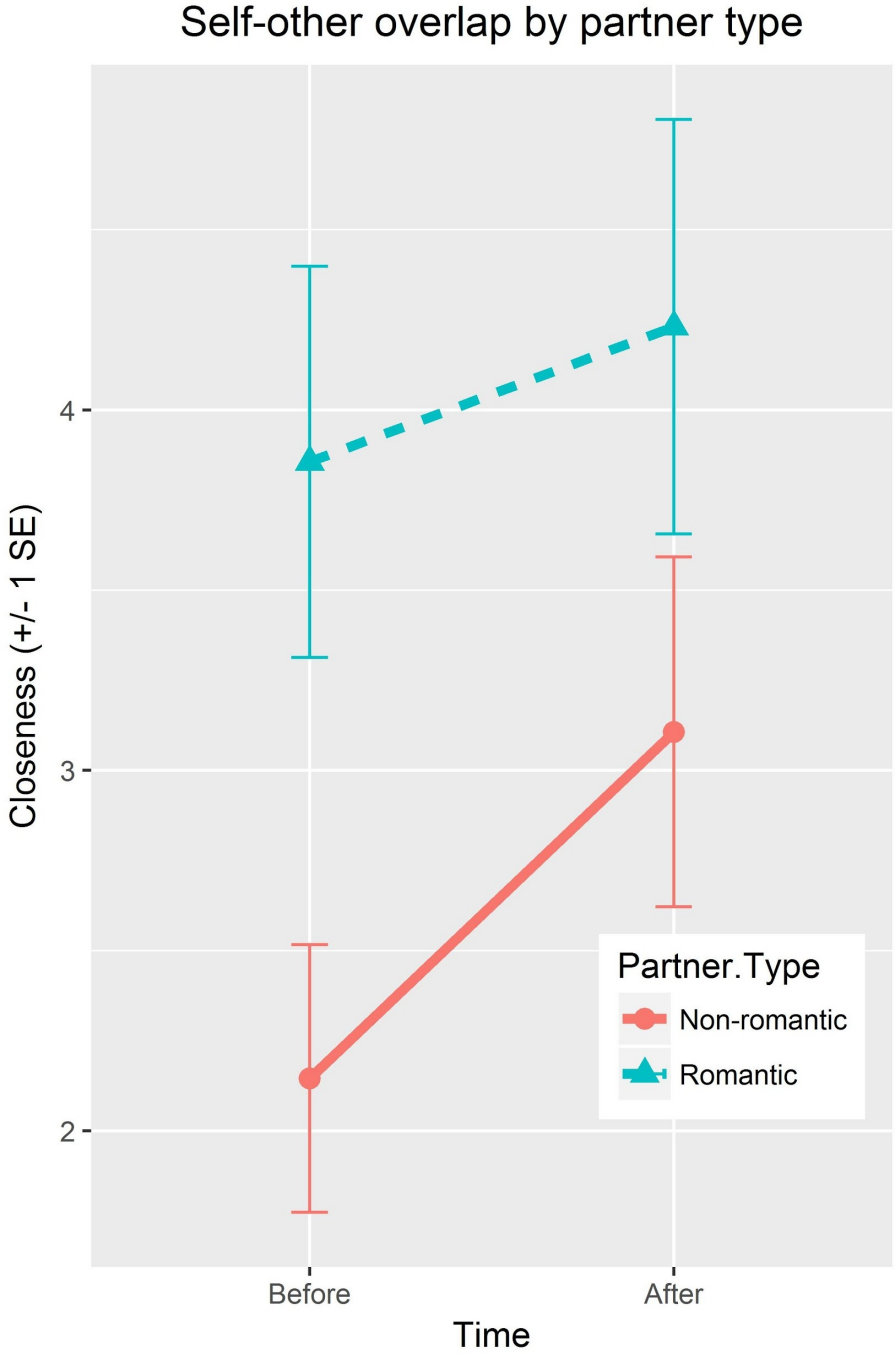
Interaction of closeness around OM by partner type.

There was a main effect of time such that, following OM, all participants felt closer to their partner (*t*(205) = 8.3, *p* < .001, *CI* = 1.2 to .7, Hedges = .38). The addition of partner type improved the prediction of closeness beyond time (preOM, postOM) alone (AIC = 1463.4 vs 1411.0, BIC = 1483.5 vs. 1439.2, *L*(2,7) = 56.4, $R_{marginal}^{2}$ = .21). Specifically, time and partner type interacted (*t*(205) = 3.6, *CI* = .27 to .91, $R_{marginal}^{2}$ = .21) as closeness increased more in non-romantic partners as compared to romantic partners (see Fig 1). To ensure that the model fit did not differ by role (stroker, stroke), linear nested models adding the factor “role” also were run. The model fit did not differ significantly based on whether the person was a stroker or strokee.

There was a main effect of time such that, following OM, all participants felt more sexually aroused (*t*(205) = -8.1, *p* < .001, *CI* = -.86 to -0.53, Hedges = .56, *M(SD)* = 2.8(1.2) increased to 3.5(1.1)). The addition of partner type did not improve the prediction of sexual arousal beyond time (preOM, postOM) alone (AIC = 1269.4 vs 1272.0, *p* = .5).

Exploratory analyses examined whether OM practice frequency were associated with baseline feelings of closeness, as a way of assessing dose-response. Responses were strongly left skewed with many people practicing very frequently, so data were binned as up to “three times per week” or “four or more times a week”. This exploratory analysis was limited to the dyads who were not romantically involved (n = 104), since they would have limited opportunities to experience other positive experiences together outside OM. Those who practiced OM more frequently reported more closeness before even beginning OM than those who practiced OM less frequently (*t*(73) = -1.7, *CI* = -.85 to .06). The difference was small (*M* = 2.3 versus *M* = 1.9).

## Discussion

In this study, 125 dyads completed one session of Orgasmic Meditation (OM). They reported feeling higher closeness after OM as compared to before OM. An interaction demonstrated that the increase in closeness was most pronounced for dyads who were not romantic partners. This pattern of results is consistent with the use of OM for causing increases in closeness that appear useful in a variety of immediate tasks. Sexual arousal did not vary as a function of partner type. The relationship between relationship type and closeness are not likely due simply to having greater sexual arousal with a novel sexual partner (non-romantic) or a regular, trusted sexual partner (romantic). The pattern of results also is consistent with the functional, self-expansion, or hedonic touch, hypotheses, that shared, intensely positive experiences like genital touch will increase interpersonal closeness. The larger improvement in closeness for non-romantic partners was not necessarily predicted by the self-expansion hypothesis, but appears consistent with it.

Exploratory analysis was consistent with the idea that practicing OM more frequently might offer some more sustained increase in relationship closeness beyond after the OM session alone. Effects of increased closeness from non-OM sex partners appears to extend for days [85]. Depending on the mechanism, OM effects also might extend beyond the immediate time-frame of the laboratory.

The interaction of time and relationship status on closeness appears to reflect more than a ceiling effect. The range of the closeness rating scale is 1 to 7. Romantic partners after OM still averaged around 4.3 after OM, which is 2.7 units from the maximum ratings. Also, these ratings indicate higher closeness than the average closeness reported for friends (*M* = 2.8), family members (*M* = 2.7) [86], and comparable ratings to dating partners (*M* = 4.8) and spouses (*M* = 4.9) [87] in trait-based studies.

Our primary question regarded the extent to which partner status affects how sexual touch impacts relationship closeness. That said, we observed a strong main effect of relationship closeness for OM. While such increases in closeness may not be specific to OM (e.g., may occur for non-sexual touch), they do appear fairly strong compared to other interventions constructed to improve relationship closeness in the literature. For example, meta-analyses of marital and family interventions have notoriously weak effects in general (d < .5) [88]. Those which specifically examine closeness and intimacy regularly fare worse. For example, in a study of Emotion Focused Couples Therapy [89] there was non-significant change in the primary intimacy measure, with significant changes only in exploratory analyses of the intellectual and recreational subscales. In a study of couples psychotherapy relationship intimacy in the patient increased d = .23 and for the partner d = .49, but this required “5 weekly 1-hour sessions with the individual couple and a therapist…manualized with in-session practice, handouts, and home assignments” [90]. Potentially, the current study could suggest that with techniques more similar to OM, that it is possible to increase relationship closeness in a single explicitly intimate session that focuses on an activity rather than talking about the relationship (unlike most psychotherapeutic interventions), at least in the short term. Indeed, a one-session, self-disclosure exercise between two strangers strongly increased a composite measure of relationship closeness [91], suggesting other manipulations can also increase closeness within a single session between non-romantic partners.

The clinical utility of the effect size relative to couple’s therapy appears reasonable. Specifically, an 8-week, couples mindfulness intervention was described as successful [92] with smaller increases in closeness immediately post-intervention (from 4.77 to 5.14 for men, from 4.5 to 4.91 for women) than was observed immediately after OM. Such increases would be particularly useful if they translate to lasting change, but, even if they do not, they could set couples up for positive outcomes that follow increased relationship closeness.

A specific difference between OM and other approaches in the literature is that most of these interventions are highly dependent on linguistic interactions. For couples for whom verbal communication is not strong or has not worked to increase closeness, a non-linguistic interaction may be preferable. Sexual interaction is often non-linguistic and may function to increase interpersonal closeness [93] creating potential advantages over traditional interventions for some couples. “Intimacy” has been described as a special case of closeness that includes a sexual component [94]. Self-expansion activities with a partner increased relationship satisfaction with that partner as mediated by sexual desire [71]. Some have pinpointed changes in couple’s intimacy as predictors of changes in sexual satisfaction and feelings of love [95]. Explicit motivations for sexual behaviors include increasing feelings of closeness and intimacy with the partner [96, 97]. Others report having sex to *express* closeness and intimacy with a partner [98]. A daily diary study suggested that sexual activity with a romantic partner increased relationship closeness and positive emotions for several days [85]. Notably, the reverse was not true: simply being in a positive mood did not increase the later likelihood of sexual activity in that study. Thus, some aspect of the hedonic, intense, interaction in partnered sexual activity may cause later increases in relationship closeness.

It is unclear whether the increased relationship closeness associated with OM will apply to other types of partnered interactions (i.e., external validity). For example, we believe that OM probably has different mechanisms than typical partnered sexual activity as it is explicitly structured and predictable. OM’s high level of structure helps set expectations for interactions. Thus practitioners might feel especially free to enjoy the shared experience when risks feel lower. It is unclear whether this will extend to partnered sex where interactions tend to be less structured. For example, avoidance motivation may exist to promote “prevention” and “safety” [99], which may be less important for OM than sexual activity. Sexual scripts theory, however, suggests that patterns of sexual interaction (e.g., first hugging, next male receives oral sex, etc.) might be similarly rigid and predictable [100] to OM. The generalizability to sexual contexts, including novel sexual partners, would need to be established independently. Finally, it is unclear how long such a change in closeness may last from a single OM. This is a direction for future research.

Perhaps reflecting the mixed and negative outcomes for sex with non-romantic partners, some therapies explicitly work to reduce the occurrence of non-romantic sexuality. For example, some literature on emotion-focused therapy describes sexual partners without secure romantic attachments as reflecting “promiscuity” [101], sex addiction therapists have been known to refer to non-romantic sexual relationships as “acting out” [102], and some trauma therapists have been observed to describe low commitment sexual partners as symptoms of trauma, comparing it to suicidality [e.g., 103]. While OM is clearly not sex, our data suggest pathologizing non-romantic sexuality might cause harm by reducing opportunities to connect. The external validity of this pattern of results requires exploration.

The current study has limitations. Dyads differed not only by their relationship status, but also by their individual level of relationship avoidance and anxiety (see Table 1). These baseline differences were relevant to the theories tested, such that creating non-romantic dyads with matched avoidance/anxiety would have been a poor test of the theories. An alternative would have been experimentally manipulating feelings of avoidance or anxiety prior to OM. Relatedly, dyads were required to have completed OM together. This decision was made to avoid a number of potential confounds (see Introduction). Given that no participants withdrew or reported emotional discomfort from the procedures, a next study might advance to stranger-dyads to test the generalizability of the pattern observed here. Additionally, only women received the stimulation, although the stroker could be male or female. Therefore, the generalizability of this result to male strokees remains unknown.

It remains unclear how important genital touch is for increasing relationship closeness as a part of the OM protocol. For example, stroking is experienced as erotic when it is very pleasant and low intensity, even when it occurs on the forearm or thigh [104]. A comparison condition contrasting non-genital stroking and genital stroking could determine the extent to which intimacy and self-other overlap might be promoted by non-genital stroking. Although the risk of disease transmission from OM (gloved genital touch) is negligible, some may find OM uncomfortable emotionally. Given that other areas of the body also can be experienced as erotic by varying stroking speed (see above), a slow-stroking control on non-glaborous skin will be important. The interaction with relationship type, not simply the main effect of OM, was the primary test in this study. Thus, we view this primarily as an issue of generalizability. It is unclear if the same relationship closeness would occur with non-genital touch, but genital touch appears to be one approach within which relationship differences exist.

These limitations notwithstanding, the current study has implications for how relationship closeness is approached in clinical settings. Minimally, therapists might consider the context of non-romantic sexual partners as positive, at least in the short term, with the potential that such effects could last rather than being damaging; this attitude would counter common narratives of “promiscuity” and “acting out”. Such a change could have important effects at a societal level. Non-romantic intimate partnering is common. 77.7% of women and 84.2% of men in one college campus survey reported having had a consensual, one-time sexual partner [105]. About 20% of single men and women report engaging in some form of consensually non-monogamous behaviors in their lifetimes [106]. OM itself is unlikely to be labelled “sex”, because 86.1% of college students do not consider consensually touching another’s genitals to constitute “sex” [107].

A main implication of this work is that there may be ways to improve relationship closeness which do not involve commonly appealed-to techniques such as talking, particularly about the relationship itself. Talk therapy remains the dominant method used to effect relationship change. In addition to treatment failures, talk methods also carry risks for harm [108]. Identifying other methods for improving relationships, such as touch, partnered meditation, or sexual stimulation, may yield ways to improve relationships, affect, and health that are outside the “usual candidates” of psychological research. In particular, specific behaviors within sexual interactions are more strongly associated with increased intimacy, such as kissing and cuddling [109]. Since individuals’ experiences of self-expansion varies as a function of their traits (e.g., openness to experience, agreeableness, and neuroticism), it would be useful to identify traits of those most likely to improve their closeness with OM [110]. Future studies also could identify the aspect(s) of OM most likely to promote interpersonal closeness, so experiences could be optimized to promote these gains, either as stand-alone interventions if the effects are shown to last, or as preludes to other couples-based techniques that could benefit from increased relationship closeness.
